# Voiding symptom severity varies independently from non-adrenergic prostate smooth muscle contractions in patients undergoing surgery for benign prostatic hyperplasia

**DOI:** 10.3389/fphys.2025.1612954

**Published:** 2025-05-30

**Authors:** Patrick Keller, Sheng Hu, Philip Nicola, Laurenz Berger, Alexander Tamalunas, Philipp Weinhold, Christian G. Stief, Martin Hennenberg

**Affiliations:** Department of Urology, LMU University Hospital, LMU Munich, Munich, Germany

**Keywords:** benign prostatic hyperplasia (BPH), voiding symptoms, lower urinary tract symptoms (LUTS), international prostate symptom score (IPSS), holmium laser enucleation of the prostate (HoLEP), smooth muscle contraction, human tissue

## Abstract

**Background:**

Resistance of voiding symptoms to α_1_-blockers in benign prostatic hyperplasia (BPH) has been provisionally explained by non-adrenergic prostate smooth muscle contraction. Here, we examined relationships between contractions and voiding symptoms in prostate tissues from laser-enucleation.

**Methods:**

Tissues were obtained from holmium and thulium laser enucleation. Contractions were induced by endothelin-1, U46619, noradrenaline and electric field stimulation (EFS). E_max_ values were analyzed for correlation with international prostate symptom score (IPSS), and compared to tissues from patients without surgery for BPH.

**Results:**

Noradrenaline- and EFS-induced contractions were higher with severe (IPSS 20–35) than moderate symptoms (IPSS 8–19) (E_max_ noradrenaline 66% vs 113% of KCl-induced contractions; EFS 33% vs 66%). Endothelin-1- and U46619-induced contractions were already maximum with moderate symptoms (endothelin-1 117% moderate, 135% severe; U46619 23%, 27%). Within 8–21 points, IPSS increased with E_max_ values for noradrenaline and EFS (r = 0.545, r = 0.448), but not with endothelin-1- or U46619-induced contractions. Endothelin-1-induced contractions were similar to noradrenaline-induced contractions (E_max_ endothelin-1 126% of KCl, noradrenaline 96%), and exceeded EFS- (52%) and U46619-induced contractions (25%). E_max_ values for endothelin-1 were similar between laser-enucleated patients and patients without surgery for BPH (127%), while E_max_ values for U46619 were higher in tissues from patients without surgery for BPH (59%) compared to laser-enucleated tissues.

**Conclusion:**

Symptom severity increases with α_1_-adrenergic, but not with non-adrenergic contractions in patients undergoing surgery for BPH. Endothelin-1-induced contractions are similar to noradrenaline-induced contractions. Conditions necessitating BPH surgery may not necessarily depend on α_1_-adrenergic tone, but may involve non-adrenergic contractions or factors beyond contraction.

## 1 Introduction

Voiding symptoms in benign prostatic hyperplasia (BPH) are among the most common conditions affecting elderly men and are primarily attributed to urethral obstruction caused by increased prostate smooth muscle tone and prostate enlargement (Lepor, 2004). The first-line option in non-conservative treatment are α_1_-adrenoceptor antagonists (α_1_-blockers), which are thought to alleviate symptoms by reducing prostate smooth muscle contraction (Gravas et al., 2023; Hennenberg and Michel, 2024). However, symptom relief remains incomplete in most patients, and the number of non-responders exceeds 30% (Hennenberg and Michel, 2024). Surgery is the last treatment option for patients who have exhausted all medical options, or is performed when complications become imminent or recurrent (Gravas et al., 2023). Transurethral resection of the prostate (TURP) remains the gold standard for BPH surgery, but is increasingly being replaced by laser enucleation in centers with appropriate expertise (Magistro and Stief, 2020; Gravas et al., 2023).

Based on quantification by the international prostate symptom score (IPSS) (Gratzke et al., 2015; Sandhu et al., 2024), voiding symptoms may be classified to mild or bothersome (0–7 points), moderate (8–19 points) and severe stages (20–25 points) (Partin et al., 2020). α_1_-Blockers are recommended for moderate to severe symptoms, whereas patients undergoing surgery for BPH are mostly unresponsive to medical treatment. The limited efficacy of α_1_-blockers and medication-refractory voiding symptoms have been provisionally explained by non-adrenergic prostate smooth muscle contractions (Hennenberg et al., 2014; Hennenberg and Michel, 2024). In prostate tissues from patients undergoing surgery for prostate cancer (PCa) and without prior surgery for BPH, endothelin-1 and thromboxane A_2_ induce full contractions, potentially maintaining increased smooth muscle tone and symptoms even despite α_1_-blocker treatment. However, non-adrenergic prostate smooth muscle contractions in patients with medication-refractory symptoms remain unexplored, and their relationship with symptom severity are unknown.

Recently, laser-enucleated prostate tissues from BPH surgery were used for the first time to study adrenergic and neurogenic prostate smooth muscle contractions (Keller et al., 2025). Compared to TURP-derived tissues, contractility in laser-enucleated tissues appears largely preserved, possibly due to reduced surgical traumatization (Keller et al., 2025). Here, we examined endothelin-1- and thromboxane A_2_-induced contractions in tissues from holmium and thulium laser enucleation of the prostate (HoLEP, ThuLEP), and the relationships of non-adrenergic and adrenergic contractions with symptom severity.

## 2 Materials and methods

### 2.1 Structure of the study

This study was carried out in accordance with the Declaration of Helsinki of the World Medical Association and has been approved by the ethics committee of the Ludwig-Maximilians University, Munich, Germany (approval number 22–0,608, from 08–10–2022). Informed consent was obtained from all individual participants included in the study. Samples and data were collected and analyzed pseudonymized. Laser-enucleated prostate tissue from surgery for BPH was obtained from patients without prior catheterization for urinary retention and from whom IPSS data were available from preoperative anamneses. These tissues were used for concentration-response curves for endothelin-1 and U46619 in organ bath experiments, with subsequent calculation of E_max_ values, and grouping and correlation analyses with IPSS. Concentration response curves and E_max_ values for noradrenaline and EFS were reanalyzed from data of a previous study with laser-enucleated tissues (Keller et al., 2025), but only tissues from patients without catheterization and from whom IPSS data were available were included here, for comparison to non-adrenergic contractions assessed in the current study. For analyses of this study, only tissue from patients with an IPSS ≥8 was included, according to the suggested staging to moderate symptoms (IPSS 8–19) and severe symptoms (20–35), while scores <8 are rare in patients undergoing surgery for BPH. E_max_ values for endothelin-1- and U46619-induced from this study were finally compared to E_max_ values from tissues obtained from radical prostatectomy (rPx) for prostate cancer in our previous studies published 2018–2024 (including 20 studies with endothelin-1, and 19 with U46619) (Hennenberg et al., 2017b; Hennenberg et al., 2018; Herlemann et al., 2018; Yu et al., 2018a; Yu et al., 2018b; Yu et al., 2019a; Yu et al., 2019b; Li et al., 2020a; Li et al., 2020b; Wang et al., 2020; Huang et al., 2021; Tamalunas et al., 2021b; Tamalunas et al., 2021a; Huang et al., 2022a; Huang et al., 2022b; Tamalunas et al., 2022a; Tamalunas et al., 2022b; Liu et al., 2023; Hu et al., 2024; Liu et al., 2024). Patients with prior TURP or laser enucleation were excluded from tissue collection from rPx, so that this population may reflect patients with low-symptom, uncomplicated BPH. Typically, 80% of patients with prostate cancer show BPH (Alcaraz et al., 2009; Orsted and Bojesen, 2013), and the age of patients undergoing rPx at our department averages out at 66 ± 7 years (Grabbert et al., 2018), when the prevalence of histological BPH ranges between 60% and 70% (Lepor, 2004).

### 2.2 Tissues from holmium and thulium laser enucleation of the prostate

HoLEP and ThuLEP were performed in a three-lobe technique as recently described (Keller et al., 2025). Following extraction of morcellated tissues from the bladder, tissue samples were immediately transferred to Custodiol® solution (Köhler, Bensheim, Germany) for transport, interim storage and selection of tissue shreds used for organ bath experiments. Organ bath experiments were started within 2 h following extraction of morcellates from the bladder.

### 2.3 Data from tissues from radical prostatectomy

E_max_ values for endothelin-1- and U46619-induced contractions of prostate tissues from rPx for prostate cancer were compiled from control groups in our 20 previous studies with endothelin-1 and U46619 in these tissues, which were published from 2017–2024 (Hennenberg et al., 2017b; Hennenberg et al., 2018; Herlemann et al., 2018; Yu et al., 2018a; Yu et al., 2018b; Yu et al., 2019a; Yu et al., 2019b; Li et al., 2020a; Li et al., 2020b; Wang et al., 2020; Huang et al., 2021; Tamalunas et al., 2021b; Tamalunas et al., 2021a; Huang et al., 2022a; Huang et al., 2022b; Tamalunas et al., 2022a; Tamalunas et al., 2022b; Liu et al., 2023; Hu et al., 2024; Liu et al., 2024). E_max_ values were collected from each single experiment, with most of these values representing the mean of two samples from the same prostate as double determination in the same experiment. Data were reanalyzed by curve fitting if concentration response curves included other ranges as experiments in this study, to align E_max_ values from previous studies with conditions applied in the current study. Values were obtained from the control groups and were consequently obtained in the presence of solvents (mostly dimethylsulfoxid, DMSO) in varying amounts. Tissues were collected from periurethral zones. Conditions for interim storage and transport were similar to conditions for tissues from laser enucleation in this study, with the exception that tissues from rPx were macroscopically inspected and sampled by pathologists.

### 2.4 Organ bath experiments

Tissue shreds with sizes for organ bath experiments (approximately 6 × 3 × 3 mm) required for organ bath experiments were either selected directly and without further cutting from the macerations, or prepared by cutting largest available shreds (Keller et al., 2025). Tissue strips were mounted in organ baths containing four chambers per device (model 720 M, Danish Myotechnology, Aahus, Denmark), each filled with 10 mL Krebs-Henseleit solution (37°C, pH 7.4) continuously gassed with carbogen (95% O_2_ and 5% CO_2_) (Hu et al., 2024; Keller et al., 2025). After adjustment of a stable pretension of 4.9 mN within 45 min as recently described (Hu et al., 2024), tissues were contracted by 80 mM KCl, by the addition of a 2 M KCl solution. As soon as a maximum contraction was obtained, chambers were washed three times with normal Krebs-Henseleit solution to remove the high molar KCl, until a new stable baseline was reached again. Subsequently, cumulative concentration response curves for endothelin-1 or the thromboxane A_2_ receptor agonist U46619 (both from Enzo Life Sciences, Lörrach, Germany) were constructed. Endothelin-1 was dissolved in dimethylsulfoxide (DMSO) and stock solutions (0.4 mM) were stored at −20°C until use, as small aliquots so that repeating freezing and thawing cycles were avoided. U46619 was dissolved in ethanol, and stock solutions (10 mM) were stored at −80°C until use. Only one concentration response curve was recorded per tissue strip. Strips were intuitively allocated to examination with endothelin-1 or U46619. Channels showing no reaction to KCl were not further examined, and included to analyses by rating as 0 mN at each agonist concentration. Agonist- and EFS-induced contractions are expressed as percentage of 80 mM KCl-induced contractions to correct variations and individual heterogeneities, and variables such as strip size or smooth muscle content.

Tissues from 23 patients were examined with endothelin-1 (12 with IPSS 8–19, 11 with IPSS 20–35), tissues from 38 patients with U46619 (20 with IPSS 8–12, 18 with IPSS 20–35), tissues from 27 patients with noradrenaline (10 with IPSS 8–19, 17 with IPSS 20–35), and tissues from 21 patients with EFS (9 with IPSS 8–19, 12 with IPSS 20–35). The majority of experiments included two to four strips per tissue, while single determinations with only 1 strip per tissue included one experiment with endothelin-1 and moderate symptoms, one experiment with endothelin-1 and severe symptoms, three experiments with U46619 and moderate symptoms, three experiments with noradrenaline and moderate symptoms, one experiment with EFS and moderate symptoms, and two experiments with EFS and severe symptoms.

E_max_ values, EC_50_ values for agonists, and frequencies inducing 50% of the maximum EFS-induced contraction (Ef_50_) were calculated separately for each single experiment by curve fitting, using GraphPad Prism 6 (GraphPad Software Inc., San Diego, CA, United States). The software sends error messages, if curve fitting is not possible, or if results from curve fitting are suspected as “ambiguous”. In addition, values from curve fitting were checked manually for plausibility, as recommended in the “GraphPad Curve Fitting Guide” (GraphPad Software Inc.). Ambiguous and non-plausible values occurred in one experiment with endothelin-1 (moderate symptoms) and two experiments with U46619 (severe symptoms), which were replaced by the highest applied agonist concentrations and the contractions induced by this concentration for further analysis. Downhill parts of concentration response curves at high agonist concentrations, which precluded curve fitting or plausible results had to be excluded from curve fitting in two experiments with U46619 and moderate, and again in two experiments with U46619 and severe symptoms. Curve fitting was not possible with tissues showing no contractions, so that E_max_ values for these tissues were set to 0 mN, and EC_50_ values were not included.

### 2.5 Statistical analyses

Data in concentration and frequency response curves are means with standard deviation (SD). Single values in scatter plots are means from all strips examined per tissue. Data in the text are reported as means with 95% confidence intervals (95% CI). Distribution of E_max_, EC_50_ and Ef_50_ values was assessed by the D'Agostino and Pearson omnibus normality test (alpha = 0.05). Data sets containing at least one group without Gaussian distribution were analyzed by non-parametric tests, while parametric tests were applied if all groups showed Gaussian distribution. If group sizes did not allow normality analyses, the data distribution was estimated using scatter plots. Comparison of whole frequency and concentration response curves was performed by two-way analysis of variance (ANOVA), without multiple comparison. E_max_, EC_50_ and Ef_50_ values between two groups (i.e., different IPSS groups) were compared by unpaired, two-tailed Mann Whitney test if data were not normally distributed in at least one of both groups, and by unpaired, two-tailed t-test if data were normally distributed in both groups. E_max_ values for endothelin-1, U46619, noradrenaline and EFS in laser-enucleated tissues were compared by one-way ANOVA with Holm-Sidak’s multiple comparisons test for a data set with Gaussian distribution in each group, and by one-way ANOVA with Dunn’s multiple comparisons test in data sets containing groups without Gaussian distribution. Comparisons of previously reported E_max_ values from rPx tissues to E_max_ values in the current study were performed by Dunn’s multiple comparison after one-way ANOVA with Kruskal Wallis test, allowing comparison of multiple groups without normal distribution with a shared control group. *P* values < 0.05 were considered significant. *P* values ≥0.05 are not indicated. Relationships between E_max_ values and IPSS were analyzed by calculation of Pearson correlation coefficients (r). All data analyses were performed using GraphPad Prism 6. The present study has an exploratory design, as typical features of a hypothesis-testing study are lacking, including a clear preset study plan, blinding, or biometric calculation of group sizes (Michel et al., 2020). Consequently, *p* values are descriptive, but not hypothesis-testing (Michel et al., 2020). The formation of group sizes was not driven by power calculations, but 10 independent experiments per series were consistently found sufficient to detect biologically relevant differences or to detect drug effects in our previous organ bath experiments.

## 3 Results

### 3.1 Noradrenaline-induced contractions

**FIGURE 1:**
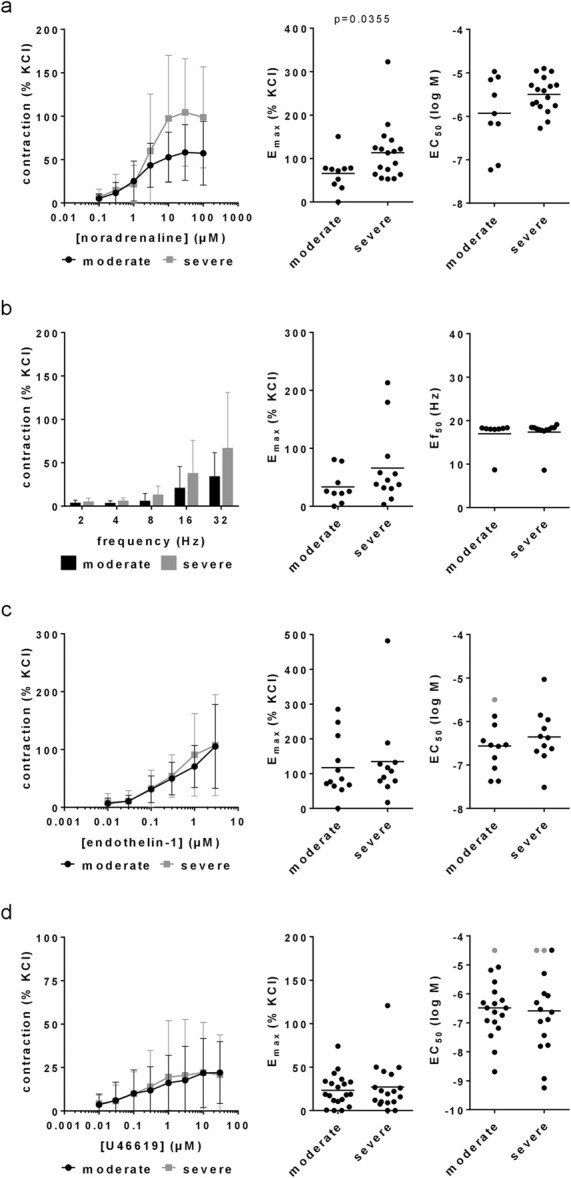
Agonist- and EFS-induced contractions in laser-enucleated prostate tissues. Concentration or frequency response curves for noradrenaline **(a)**, EFS **(b)**, endothelin-1 **(c)** and U46619 **(d)** were constructed with prostate tissues from HoLEP and ThuLEP for BPH. Data were grouped for moderate (IPSS 9–19) and severe symptoms (IPSS 20–35). Shown are means ± standard deviation (SD) in concentration and frequency response curves, and all single values for E_max_, EC_50_ and Ef_50_ calculated by curve fitting (each value representing one prostate tissue, examined by single or multiple determinations) together with means (bars). Concentration and frequency response curves were compared by two-way ANOVA. E_max_ and Ef_50_ values were compared by unpaired, two-tailed t-test if data were normally distributed in both groups (i.e., EC_50_ values in **(a)**, **(c)** and **(d)**), and by unpaired, two-tailed Mann Whitney test if data were not normally distributed in at least one of both groups (all others). *P* values ≥0.05 are not shown. Values labelled by grey color could not be calculated by curve fitting as contractions occurred only with highest applied concentrations of U46619 or were not at maximum with the highest applied concentration of endothelin-1, so that these EC_50_ values were manually set to 4.5 for U46619 or 5.5 for endothelin-1. E_max_ values from tissues showing no contraction at all are included (corresponding to an E_max_ of 0% of KCl), but plausible EC_50_ values from these experiments can not be calculated or assumed, so that these were excluded (1 value in the moderate groups for noradrenaline and EFS, two values per group for U46619, one value in the moderate group for endothelin-1).

Noradrenaline induced concentration-dependent contractions, which were higher in tissues from patients with severe symptoms compared to tissues from patients with moderate symptoms (Figure 1a). The E_max_ for noradrenaline-induced contractions amounted to 66% [37%–94%] of KCl-induced contractions in tissues from patients with an IPSS of 8–19, but to 113% [79%–147%] of KCl-induced contractions with an IPSS of 20–35 (Figure 1a). The EC_50_ (logM) for noradrenaline amounted to −5.929 [-6.575 to −5.284] with an IPSS of 8–19, and to −5.494 [-5.702 to −5.286] with an IPSS of 20–35 (Figure 1a).

### 3.2 EFS-induced contractions

EFS induced frequency-dependent contractions, which were higher in tissues from patients with severe symptoms compared to tissues from patients with moderate symptoms (Figure 1b). The E_max_ for EFS-induced contractions amounted to 33% [12%–55%] of KCl-induced contractions with an IPSS of 8–19, but to 66% [25–107] with an IPSS of 20–35 (Figure 1b). The Ef_50_ amounted to 17 Hz [14–20 Hz] with an IPSS of 8–19, and again to 17 Hz [16–19 Hz] with an IPSS of 20–35 (Figure 1b).

### 3.3 Endothelin-1-induced contractions

Endothelin-1 induced concentration-dependent contractions, which were similar in tissues from patients with moderate and severe symptoms (Figure 1c). The E_max_ for endothelin-1-induced contractions amounted to 117% [62%–172%] of KCl-induced contractions in tissues from patients with an IPSS of 8–19, and to 135% [52%–217%] of KCl-induced contractions with an IPSS of 20–35 (Figure 1c). The EC_50_ (logM) for endothelin-1 amounted to −6.565 [-6.962 to −6.167] with an IPSS of 8–19, and to −6.355 [-6.777 to −5.933] with an IPSS of 20–35 (Figure 1c).

### 3.4 U46619-induced contractions

U46619 induced concentration-dependent contractions, which were similar in tissues from patients with moderate and severe symptoms (Figure 1d). The E_max_ for U46619-induced contractions amounted to 23% [15%–32%] of KCl-induced contractions in tissues from patients with an IPSS of 8–19, and to 27% [13%–41%] of KCl-induced contractions with an IPSS of 20–35 (Figure 1d). The EC_50_ (logM) for U46619 amounted to −6.484 [-6.996 to −5.973] with an IPSS of 8–19, and to −6.585 [-7.361 to −5.808] with an IPSS of 20–35 (Figure 1d).

### 3.5 Correlations of e_max_ values with IPSS

**FIGURE 2:**
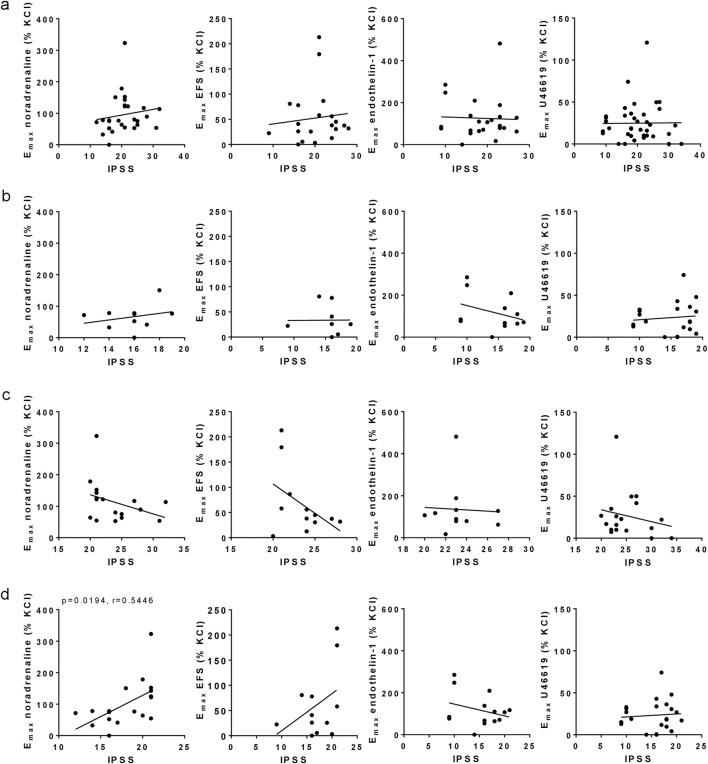
Correlation analyses for E_max_ values with IPSS. E_max_ values for noradrenaline-, EFS-, endothelin-1- and U46619-induced contractions in laser-enucleated prostate tissues were analyzed for correlation with IPSS scores in the same patients. Separated analyses were performed for the complete study populations (IPSS 8–35) **(a)**, for moderate symptoms (IPSS 8–19) **(b)**, severe symptoms (IPSS 20–35) **(c)**, and for a modified IPSS range (IPSS 8–21) **(d)**. Shown are all data, together with r and p values from Pearson correlation analyses. *P* values ≥0.05 are not shown.

No correlations were observed between IPSS with E_max_ values for noradrenaline, EFS, endothelin-1 or U46619 in the entire study population (i.e., including IPSS values from 8–35), apart from small increases of IPSS with noradrenaline- and EFS-induced contractions (r = 0.1488, r = 0.1149) (Figure 2a). The IPSS did not increase with E_max_ values after grouping for moderate symptoms (8–19 points) (Figure 2b), or for severe symptoms (20–35 points) (Figure 2c). An accumulation of high E_max_ values for noradrenaline and EFS was observed in an IPSS range of 20–21 points (Figure 2a), so that an additional analysis with a cut-off point of 21 IPSS points instead of 19 points was performed (Figure 2d). Within a range of 8–21 points, the IPSS increased with E_max_ values for noradrenaline (r = 0.5446) and EFS (r = 0.4483), but not with E_max_ values for endothelin-1 or U46619 (Figure 2d).

### 3.6 Comparison of non-adrenergic, adrenergic and neurogenic contractions

**FIGURE 3:**
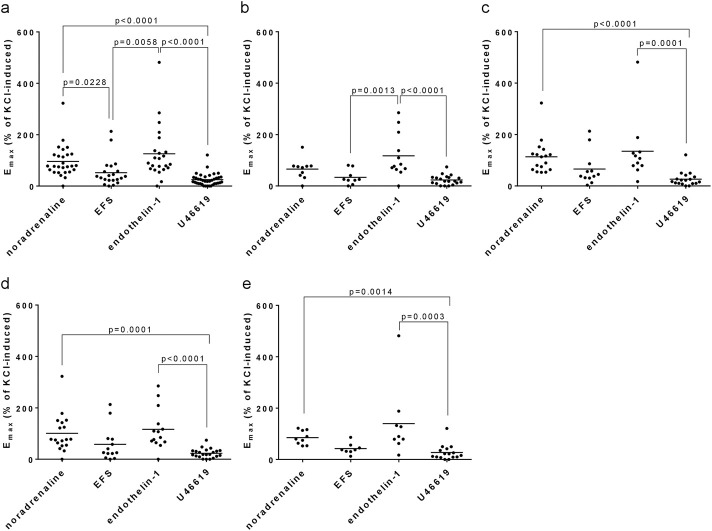
Comparison of E_max_ values for agonist- and EFS-induced contractions. E_max_ values for noradrenaline, EFS, endothelin-1 and U46619 in laser-enucleated tissues were compared with each other. Separated comparisons were performed for the complete study populations (IPSS 8–35) **(a)**, for moderate symptoms (IPSS 8–19) **(b)**, severe symptoms (IPSS 20–35) **(c)**, and for modified IPSS ranges in **(d)** (IPSS 8–21) and **(e)** (IPSS 22–35). Shown are all data, together with *p* values from one-way ANOVA with Holm-Sidak’s multiple comparison for data showing Gaussian distribution (D'Agostino and Pearson omnibus normality test) **(b)**, or with Dunn’s multiple comparison of data sets without Gaussian distribution (all others). *P* values ≥0.05 are not shown.

E_max_ values for endothelin-1 were similar to E_max_ values for noradrenaline in the entire study population (IPSS 8–35) (Figure 3a), and in subgroups with an IPSS of 8–19 (Figure 3b), of 20–35 (Figure 3c), of 8–21 (Figure 3c) and an IPSS of 22–35 (Figure 3d). E_max_ values for endothelin-1 and noradrenaline were higher than E_max_ values for EFS and U46619, in the entire study population and within all subgroups (Figure 3). Specifically, E_max_ values for endothelin-1, U46619, EFS and noradrenaline amounted to 126% of KCl-induced contraction [81%–170%], 25% [17%–32%], 52% [27%–77%] and 96% [71%–120%] in the entire study population, 117% [62%–172%], 23% [15%–32%], 33% [12%–55%] and 66% [37%–94%] with an IPSS of 8–19, 135% [52%–217%], 26% [13%–40%], 66% [25%–107%] and 113% [79%–147%] with an IPSS of 20–35, 117% [71%–162%], 23% [15%–31%], 58% [18%–99%] and 101% [65%–137%] with an IPSS of 8–21, and 140% [34%–245%], 27% [11%–43%], 42% [24%–60%] and 85% [64%–106%] with an IPSS of 22–35.

### 3.7 Comparison of non-adrenergic contractions in laser-enucleated tissues and in tissues from rPx

**FIGURE 4:**
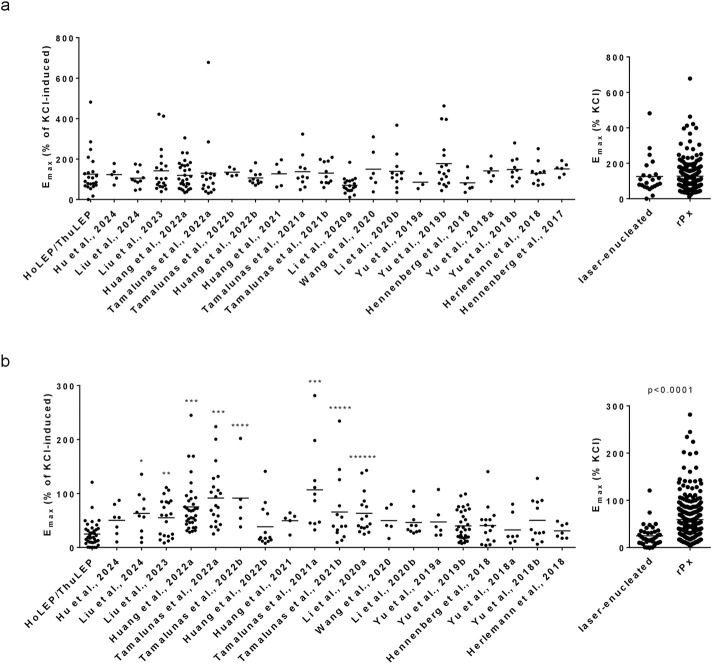
Comparison of E_max_ values for non-adrenergic contractions in laser-enucleated and prostatectomized tissues. E_max_ values for endothelin-1 **(a)** and U46619 **(b)** in prostate tissues from HoLEP and ThuLEP were compared to E_max_ values in prostate tissues from rPx for prostate cancer in previous studies, published from 2017 to 2024. Shown are single values from each experiment, together with means and p values from one-way ANOVA with Dunn’s multiple comparison (*p = 0.023, **p = 0.0108, ***p > 0.0001, ****p = 0.0144, *****p = 0.0495, ******p = 0.0014). *P* values ≥ 0.05 are not shown.

E_max_ values for endothelin-1 were similar between laser-enucleated tissues and tissues from rPx for PCa in 20 previous studies (Figure 4a). The E_max_ for endothelin-1 in laser-enucleated tissues amounted to 126% of KCl-induced contraction [81%–170%], while the lowest and highest average E_max_ values in rPx tissues amounted to 70% [52%–89%] and 178% [112%–243%] (Figure 4a). If compiled to one single group, the E_max_ value for endothelin-1 in rPx tissues (n = 216) amounted to 127% of KCl-induced contraction [116%–138%] (Figure 4a).

E_max_ values for U46619 were similar between laser-enucleated tissues and tissues from rPx for PCa in 11 previous studies (Figure 4b). In eight previous studies with rPx tissues, E_max_ values for U46619 were higher as in laser-enucleated tissues (Figure 4b). The E_max_ for U46619 in laser-enucleated tissues amounted to 25% [17%–32%], while the lowest and highest average E_max_ values in rPx tissues amounted to 31% [18%–43%] and 107% [50%–164%] (Figure 4b). If compiled to one single group, the E_max_ value for U46619 in rPx tissues (n = 251) amounted to 59% of KCl-induced contraction [53%–65%], which was higher compared to laser-enucleated tissues (Figure 4b).

## 4 Discussion

Our findings suggest that non-adrenergic contractions are constant across all IPSS stages in patients needing surgery for BPH, whereas adrenergic contractions vary with symptom severity. Endothelin-1-induced contractions were similar in strength to noradrenaline-induced contractions in laser-enucleated tissues, and to endothelin-1-induced contractions in tissues from prostate cancer patients without prior surgery for BPH. Thromboxane-induced contractions were overall weak, suggesting a minor role in prostate smooth muscle tone or urethral obstruction of BPH patients requiring surgery. Findings of this study may help to understand why α_1_-blockers are ineffective in these patients.

Non-adrenergic contractions have been previously studied in tissues from rPx, and were suspected to account for α_1_-blocker-resistant voiding symptoms in BPH (Hennenberg et al., 2013; Hennenberg et al., 2014; Hennenberg and Michel, 2024). α_1_-Blockers are the first-line medical treatment for voiding symptoms, and are believed to improve symptoms by inhibition of α_1_-adrenergic prostate smooth muscle contraction (Gravas et al., 2023; Hennenberg and Michel, 2024). Non-adrenergic prostate smooth muscle contractions are insensitive to α_1_-blockers (Hennenberg et al., 2017a), possibly maintaining urethral obstruction and symptoms elevated despite α_1_-blocker treatment. Endothelin-1-induced contractions are not additive with noradrenaline, potentially compensating for a lacking adrenergic tone (Hennenberg et al., 2013; Hennenberg et al., 2017a). Although α_1_-blockers undoubtedly improve the situation in a number of patients, their overall efficacy is limited. In placebo run-in controlled studies, α_1_-blockers reduced IPSS by 30%–50% and increased the Q_max_ by 20%–40% (Oelke et al., 2013). Open-label studies without a run-in phase reported IPSS reductions of up to 50% and Q_max_ increases of up to 40% (Michel et al., 1998; Djavan et al., 2004; Oelke et al., 2013). Large-scale trials demonstrated IPSS decreases from −3.8 to −7 points and Q_max_ increases between 0.7 and 3.77 mL/s (McConnell et al., 2003; Roehrborn et al., 2010; Chapple et al., 2011). However, even placebos reduced the IPSS by over 30% and enhanced the Q_max_ by up to 15% (Hennenberg and Michel, 2024). Exemplarily, the IPSS was reduced −7.0, −6.7 and −4.7 points with silodosin, tamsulosin and placebo, while the Q_max_ was improved by 3.77, 3.53 and 2.53 mL/s in a phase III trial (Chapple et al., 2011). A meta-analysis of 25 trials found IPSS reductions of 4.4 points and Q_max_ increases of 0.8 mL/s by placebos, with the strongest effects in studies where treatment responses were expected to be highest (Eredics et al., 2017). In 30%–35% of patients, IPSS reductions do not exceed 25%, leaving up to 69% dissatisfied, contributing to discontinuation rates about 65% within 12 months (Chapple et al., 2011; Hennenberg and Michel, 2024). Poor adherence may increase hospitalization rates and the likelihood of BPH-related surgery (Cindolo et al., 2015a; Cindolo et al., 2015b).

In our study population, 67% of laser-enucleated patients had received α_1_-blockers until surgery, and all included patients had an IPSS ≥8. Surgery for BPH is not only performed if drug treatment becomes insufficient or is declined by patients, but typically if complications become imminent or recur (Oelke et al., 2013; Magistro and Stief, 2020; Gravas et al., 2023). Thus, symptoms in these patients are not necessarily medication-refractory, but α_1_-blockers were ultimately ineffective in our study cohort, and BPH progression and voiding symptoms were more pronounced than in prostate cancer patients without prior surgery for BPH. To the best of our knowledge, our study is the first addressing non-adrenergic prostate smooth contractions and their relationship with symptom severity in patients undergoing surgery for BPH.

Our results may contribute to understanding why α_1_-blockers are ineffective in these patients. Symptom severity increased with norepinephrine-induced contractions, although all patients required surgery despite α_1_-blocker treatment. Therefore, we conclude that α_1_-adrenergic contractions are not necessarily decisive for obstruction, complications, or the need for surgery. In contrast, endothelin-1-induced contractions were constant across all symptom severities, implying that endothelin-mediated tone may contribute more to complications and surgical indications than adrenergic contractions. This could lead to concepts speculating that adrenergic contractions contribute to symptoms, while non-adrenergic contractions contribute to complications and surgical indications. However, tissues from both laser-enucleated and prostatectomized patients showed similar responses to endothelin-1 and similar responses to noradrenaline, suggesting that smooth muscle tone may not be the primary determinant of disease severity, at least in laser-enucleated patients. Instead, hyperplastic growth, particularly in the periurethral zone, or glandular rather than stromal hyperplasia may be more relevant in patients requiring surgery for BPH. However, tissue heterogeneity in BPH is high and insufficiently understood (Strand et al., 2017), and our conclusions remain speculation unless evidence proving causal links become available.

It should be noted that our contraction measurements were performed *ex vivo*, using exogenous agonists *in vitro*, which does not allow any conclusions to be drawn about the presence or activity of these agonists *in vivo*. Clinically, responses to α_1_-blockers are proportional to the percentage of smooth muscle cells in the prostate, and may be consequently insufficient in patients with predominant glandular hyperplasia (Strand et al., 2017). Thromboxane-induced contractions appear too weak to be relevant for symptoms or complications, as they were markedly lower than adrenergic and endothelin-1-induced contractions. Our study focussed on contractile responses induced by agonists, but did not examine contributions of smooth muscle relaxing factors, which may include neuronally released nitric oxide and others (Takeda et al., 1995). Impaired release of relaxing factors may promote a contracted state, which may account for the observed differences between IPSS groups in our study. Smooth muscle relaxing factors can affect contractions by different agonists differently, which is better understood for the urinary bladder than for the prostate (Erdogan and Michel, 2022; Hennenberg and Michel, 2024). In addition, prostate fibrosis may have contributed to the different contractilities in our study. Prostatic fibrosis increases the tissue stiffness and may contribute to urethral obstruction by the periurethral zone (Rodriguez-Nieves and Macoska, 2013; Bushman and Jerde, 2016). Tissue remodeling by fibrosis may replace smooth muscle cells by deposition of extracellular matrix compounds in the prostate (Rodriguez-Nieves and Macoska, 2013; Bushman and Jerde, 2016), which may result in a loss of contractility. Thus, the observed group differences may reflect differences in BPH-related fibrosis, alterations in relaxing signaling, or contributions of mixed factors, which merits further investigation.

Laser-enucleated tissues have only recently been investigated for contractile responses, but this was limited to adrenergic and EFS-induced contractions (Keller et al., 2025). Compared to TURP tissues, laser-enucleated tissues exhibited stronger contractions and a lower proportion of non-responders to contractile stimuli (Keller et al., 2025), likely due to reduced surgery-related tissue trauma and qualifying them as a new model to study prostate smooth muscle contraction in highly progressed BPH. Endothelin-1- and U46619-induced contractions have been studied in previous studies using rPx tissues (Hennenberg et al., 2017b; Hennenberg et al., 2018; Herlemann et al., 2018; Yu et al., 2018a; Yu et al., 2018b; Yu et al., 2019a; Yu et al., 2019b; Li et al., 2020a; Li et al., 2020b; Wang et al., 2020; Huang et al., 2021; Tamalunas et al., 2021b; Tamalunas et al., 2021a; Huang et al., 2022a; Huang et al., 2022b; Tamalunas et al., 2022a; Tamalunas et al., 2022b; Liu et al., 2023; Hu et al., 2024; Liu et al., 2024), where endothelin-1-induced contractions were of comparable magnitude to those observed in the present study. Some earlier studies used human prostate tissues from other sources, and did not always allow reference to potassium-induced, but to adrenergic contractions. These endothelin-1-induced contractions amounted to 36%–40% of KCl-induced contractions in prostate tissues from radical cystectomy (rCx) for bladder cancer (Raschack et al., 1998), to 95% of noradrenaline-induced contraction in the same study (Raschack et al., 1998), 213% of noradrenaline in prostate tissues from rPx or rCx for cancer (Kedia et al., 2009), 203% of phenylephrine-induced contraction in tissues from rPx or rCx for cancer, but 83% of phenylephrine in tissues from suprapubic prostatectomy for BPH (without prior α_1_-blocker treatment) (Moriyama et al., 1996), 63% of noradrenaline in open prostatectomy for BPH (Takahashi et al., 2003), 76% of KCl in tissues from TURP for BPH (Ishigooka et al., 2000), and 169% of phenylephrine in tissues from TURP for bladder outlet obstruction (Mumtaz et al., 2001). Further non-adrenergic mediators induce contractions in non-human prostate tissues, but no relevant contractions in human prostate tissues, including purinergic and cholinergic agonists, dopamine, histamine or serotonin (Hennenberg et al., 2017a; Spek et al., 2021).

Potential limitations of our study include patient heterogeneity and IPSS-based assessment. The IPSS questionnaire consists of seven questions, one of which is aimed at storage symptoms attributed to overactive bladder (OAB) and not at BPH-specific complaints. As we used the scores from the full questionnaire, our findings from correlation and grouping analyses are not fully representative for voiding symptoms, but rather depict male lower urinary tract symptoms (LUTS), which are mixed in a number of patients. Patient data were collected retrospectively, after organ bath experiments, making it factually impossible to separate questionnaire components, as only the total IPSS is added to the queryable patient records, but not the original questionnaire. As analyses aiming correlation of symptoms with tissue data may benefit from separating the storage and voiding subscores, future study designs should consider this, by implementing assessments allowing prospective or retrospective separation of questionnaires. About 50% of patients with voiding symptoms also have OAB and storage symptoms, which contribute to IPSS and are α_1_-blocker-resistant, and a non-negligible number may show underactive bladder, which may contribute to voiding symptoms independent of bladder outlet obstruction (Lewis et al., 2019; Drake et al., 2020). Desobstructive surgery for BPH is performed for different indications, but true bladder outlet obstruction by invasive urodynamics is not confirmed in each patient (Bailey et al., 2015; Gravas et al., 2023), and storage symptoms have not been specifically assessed in our study cohort. In real-world practice, decisions for desobstructive surgery are often made within the framework of routine care for male LUTS and without invasive urodynamic diagnostics (Drake et al., 2020). An estimated 18%–28% of patients undergoing prostate surgery for voiding symptoms do not actually have an obstruction (Young et al., 2017). Symptoms in these patients may be primarily attributed to bladder dysfunction, and desobstructive surgery may be potentially unnecessary (Young et al., 2017; Drake et al., 2020). Although individual variation in our patient cohort may have been lower compared to the overall population of patients with BPH and voiding symptoms, as all participating patients had severely advanced BPH and required surgery, tissue heterogeneity may still have influenced our results. Phenotypic heterogeneity (e.g., including stromal, epithelial and mixed hyperplasia, or pathological conditions including inflammation, fibrosis and others) is high in BPH, but still insufficiently understood. Apart from preclinical studies, it has been supposed that lacking knowledge on phenotype heterogeneity in BPH affected study designs and outcomes in clinical trials (Strand et al., 2017), and thus represents an important aspect that needs further investigation, including tissues from laser enucleation.

## 5 Conclusion

Symptom severity increases with α_1_-adrenergic contractions, but not with non-adrenergic contractions in patients undergoing surgery for BPH. Endothelin-1-induced contractions are similar to noradrenaline-induced contractions, whereas thromboxane-induced contractions are probably too weak to contribute to bladder outlet obstruction in BPH. Conditions raising the need for surgery in BPH may not necessarily depend on α_1_-adrenergic smooth muscle tone, but may primarily involve non-adrenergic contractions or factors beyond smooth muscle contraction, including epithelial hyperplasia.

## Data Availability

The original contributions presented in the study are included in the article/supplementary material, further inquiries can be directed to the corresponding author.
